# Sofosbuvir/Velpatasvir - A Promising Treatment for Chronic Hepatitis C Virus Infection

**DOI:** 10.7759/cureus.17237

**Published:** 2021-08-16

**Authors:** Rowan Ahmed, Roaa Kareem, Nanditha Venkatesan, Rinky A Botleroo, Opemipo D Ogeyingbo, Renu Bhandari, Mallika Gyawali, Abeer O Elshaikh

**Affiliations:** 1 Internal Medicine/Family Medicine, California Institute of Behavioral Neurosciences & Psychology, Fairfield, USA; 2 Internal Medicine, All India Institute of Medical Sciences, Raipur, IND; 3 Internal Medicine, California Institute of Behavioral Neurosciences & Psychology, Fairfield, USA; 4 Medicine, California Institute of Behavioral Neurosciences & Psychology, Fairfield, USA; 5 Research, California Institute of Behavioral Neurosciences & Psychology, Fairfield, USA; 6 Public Health, Walden University, Minneapolis, USA; 7 Internal Medicine, Saint James School of Medicine, Park Ridge, USA; 8 Internal Medicine, Manipal College of Medical Sciences, Kaski, NPL

**Keywords:** sofosbuvir/velpatasvir, ribavirin, hcv hepatitis, and direct-acting antivirals agents, chronic hepatitis c virus

## Abstract

Hepatitis C virus (HCV) infection is a disease that affects millions of people worldwide and has an enormous global public health impact. Chronic HCV is a long-term infection that goes unnoticed until the virus destroys the liver enough to induce liver disease symptoms. The inadequate and poorly tolerated treatment contributes to the burden of chronic HCV. Treatments have improved over time - direct-acting antivirals (DAAs) that targeted different hepatitis C virus genomic sites have shown to be more effective and well-tolerated. Patients recover to a greater extent following a treatment regimen based on DAAs. We conducted this literature review to investigate the effectiveness of these medications in treating chronic HCV infection. Relevant articles were identified by searching PubMed and Google scholar databases. Our primary goal was to analyze the efficacy and safety of the DAA, sofosbuvir plus velpatasvir, with or without ribavirin, in cirrhotic or non-cirrhotic, naïve or previously treated, chronic HCV patients. We found that treating patients with sofosbuvir-velpatasvir for 12 weeks was highly effective with fewer adverse events, including those with compensated cirrhosis. The outcomes aided in improving HCV treatment, lowering the disease's burden and fatality rate.

## Introduction and background

Hepatitis C virus (HCV) infection is caused by an RNA virus, a member of the *Flaviviridae *family, and has six different genotypes [1]. After acute HCV infection, about 80% of patients develop a chronic, life-long disease [2]. Chronic HCV infection causes a chronic inflammatory state, with persistent viremia leading to liver fibrosis and cirrhosis in 10-20% and liver cancer in 1-5% of patients after 20-30 years [3]. Every year, 170 million people worldwide develop chronic HCV infection and are at risk of acquiring chronic liver disease, including cirrhosis and cancer [4]. In the United States (US), genotype 1 is most frequent (75%), followed by genotypes 2 and 3 at 20% and 25%, respectively [5]. The smallest group includes genotypes 4 through 6 [5].

**Figure 1:**
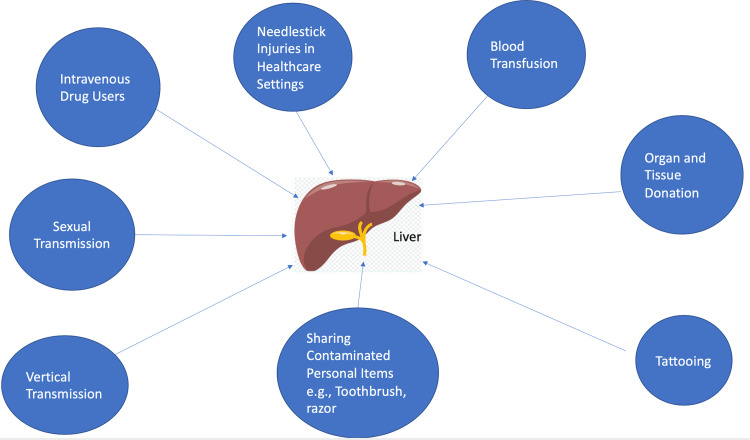
Transmission of chronic hepatitis C virus

HCV is spread predominantly by parenteral exposure to infectious blood or body fluids that contain blood [6]. Possible exposure includes injection-drug use (currently the most common mode of HCV transmission in the United States of America) and vertical transmission [6]. Although less frequent, HCV can be transmitted by personal sharing of contaminated items with infectious blood, such as razors or toothbrushes and through sex with an HCV-infected person (an inefficient mode of transmission, albeit HIV-infected men who have sex with men [MSM] have increased risk of sexual transmission) [6]. Other invasive procedures that can transmit HCV include injections, unregulated tattooing, receiving donated blood, blood products, organs (which has become uncommon since blood screening became available in the United States in 1992), and needle-stick injuries in healthcare settings [6]. The transmission of HCV is illustrated in Figure 1.

Chronic liver disease is usually insidious in people infected with HCV and progresses slowly for several decades without signs or symptoms [6]. HCV infection is not recognized until HCV-positive asymptomatic people seek blood donation or when there are elevated levels of alanine aminotransferase (ALT) during routine examinations [6]. In some individuals with symptoms, non-specific symptoms such as persistent fatigue, muscular soreness, abdominal discomfort, headache, and depression occur [7].

The American Association for the Study of Liver Diseases (AASLD) advises yearly screening for intravenous drug users and HIV-positive homosexual males [8]. For diagnosing patients with HCV infection, an anti-HCV antibody test is recommended to screen for the disease with a sensitivity of 95%, specificity of 99%, and a positive likelihood ratio of 95 [8]. When the anti-HCV antibody test is positive, qualitative measurement of HCV RNA is required to confirm current infection [8]. If the ant-HCV antibody test result is negative in patients who were exposed to HCV within the previous six months, HCV RNA should be measured every four to eight weeks for a minimum of six months, or follow up anti-HCV antibody testing should be performed every 12 weeks [8]. Patients with a positive anti-HCV antibody test result but a negative HCV RNA test result don’t have HCV infection [8]. Before starting therapy, quantitative HCV RNA testing is needed to determine the baseline viral load, and HCV genotype testing is appropriate to help guide treatment decisions [8]. All chronic HCV-infected patients are potential candidates for drug therapy [9]. Patients at risk of developing cirrhosis, as defined by a measurable hepatitis C RNA level and a liver biopsy demonstrating portal or ridging fibrosis, mild inflammation, and necrosis, should be treated [9].

**Figure 2:**
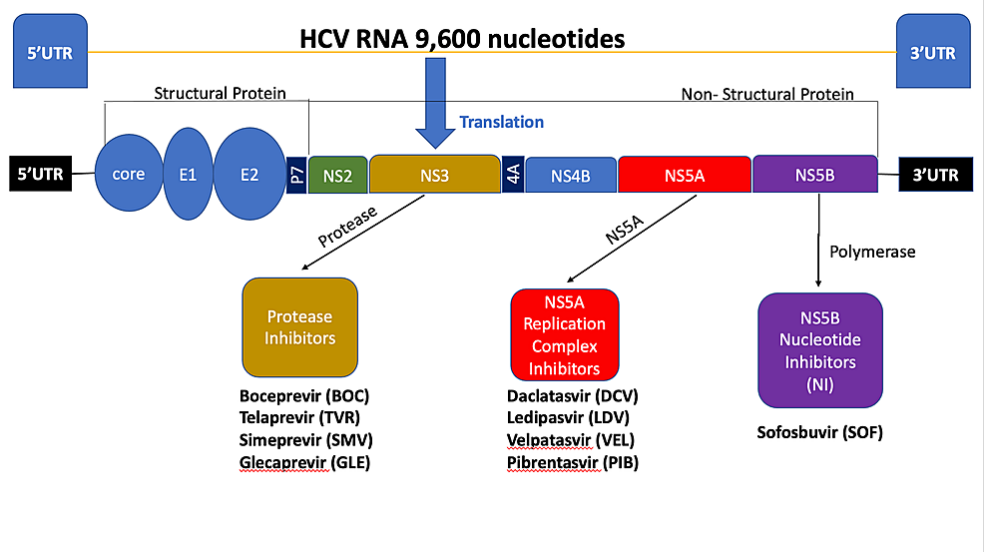
Hepatitis C virus genomic structure and the action of different direct-acting antivirals HCV: hepatitis C virus; RNA: ribonucleic acid; 5'UTR: five prime untranslated region, 3'UTR: three prime untranslated region; E1: envelope glycoprotein 1; E2: envelope glycoprotein 2; p7: ion channel; NS2: nonstructural protein 2; NS3: nonstructural protein 3; 4A: nonstructural protein 3 (NS3) protease cofactor; NS4B: nonstructural protein 4B; NS5A: nonstructural protein 5A; NS5B: nonstructural protein 5B

In 1991, the US Food and Drug Administration (FDA) approved the first treatment for HCV infection, and since then, the treatment of HCV infection has altered drastically [10]. Interferon-alpha (an immunomodulatory agent) and ribavirin (oral antiviral nucleoside analog) were the standards of care at the time, with cure rates of less than 50% with treatment necessitating self-injection, long durations of therapy and, substantial toxicity [10]. Then, more availability of culture cell models provided more profound insight into the understanding of HCV life cycle and the development of new drugs targeting non-structural HCV proteins involved in the viral replication process known as direct-acting antivirals (DAAs) [11]. These DAAs target HCV proteins, particularly the nonstructural (NS) proteins, e.g., nonstructural protein 3 and its cofactor {NS3/4A) by telaprevir, boceprevir, simeprevir; nonstructural protein 5A (NS5A) by daclatasvir, ledipasvir, and velpatasvir; and nonstructural protein 5B (NS5B) by sofosbuvir [12]. The HCV genome and different DAAS are shown in Figure 2.

The World Health Organization (WHO) modified its HCV infection screening, care, and therapy guidelines in 2016, recommending DAA-based regimens over interferon (IFN)-based regimens [13]. With the publication of the 2016 guidelines, ribavirin-free DAA regimens have increased. Regulatory bodies such as the US Food and Administration (FDA) and the European Medicines Agency (EMA) have approved several DAA regimens that successfully resolve HCV infection in more than 85% of treated people in all six major genotypes [13]. In most markets, glecaprevir-pibrentasvir (eight-week course), sofosbuvir-daclatasvir (12-week course), and sofosbuvir-velpatasvir (12-week course) are currently authorized for pan-genotypic treatment DAAs [13].

This literature review’s primary objective is to investigate the efficacy and safety of sofosbuvir/velpatasvir (SOF/VEL) in chronic HCV patients. We searched PubMed and Google Scholar and selected relevant articles, included randomized controlled trials, meta-analysis, systemic review, full-text articles, human studies only, and English articles, which took place between 2015 and 2019. 

## Review

The effectiveness of sofosbuvir/velpatasvir on chronic hepatitis C virus

Worldwide, there are six hepatitis C virus (HCV) genotypes that exist [14]. A recent systemic review estimated that globally genotype 1 (GT1) is responsible for 49% of all adult HCV infections, followed by genotype 3 (GT3) (18%), genotype 4 (GT4) (17%), genotype 2 (GT2) (11%), genotype 5 (GT5) (2%), and genotype 6 (GT6) (1%) [14]. 

The nonstructural protein 5B ( NS5B) nucleotide inhibitor sofosbuvir (SOF) is authorized for use in conjunction with other direct-acting antivirals (DAA’s) to treat HCV infection [15]. Velpatasvir (VEL) is an investigational inhibitor of the HCV NS5A protein with antiviral efficacy against all HCV genotypes (previously known as GS-5816, Gilead Sciences, Foster City, USA) [15]. The combination of VEL and SOF, administered orally and daily as a single tablet (400 mg SOF and 100 mg VEL combination) with or without ribavirin (RBV), has demonstrated excellent effectiveness in patients with all HCV genotypes [15]. To assess the efficacy of HCV treatment, we use the sustained virology response (SVR) rate [16]. SVR means that an undetectable viral load (HCV RNA <15 IU/mL) is observed at 12 weeks after completing treatment (SVR12) [16].

Feld et al. carried out a clinical trial to examine the efficacy of sofosbuvir/velpatasvir (SOF/VEL) in 740 patients with chronic HCV-GT 1, 2, 4, 5, and 6 (624 patients received treatment, and 116 patients had placebo) [1]. These patients are initially treated or untreated, and some of them had compensated cirrhosis [1]. The SVR12 was 99% (95% confidence interval [CI], 98 to >99) in the 624 patients who received SOF/VEL therapy, which was significantly higher than the prescribed 85% (p<0.001) performance target [1]. Neither of the 116 placebo patients had SVR [1]. SVR rates were similar regardless of HCV genotype ranging from 97% (95% CI, 85 to >99) to 100% (95% CI 97-100) [1]. Of the 121 patients with cirrhosis, 120 (99% [95% CI, 95 to >99) had an SVR regardless of genotype [1]. An SVR is seen in 496/501 (99%) of non-cirrhotic individuals [1].

In a meta-analysis, Ahmed et al. investigated 1427 patients who took either SOF/VEL or SOF/VEL + RBV, found that a single-tablet of SOF/VEL regimen is highly efficient in chronic HCV (GT 1-6) patients, with SVR12 rates >97% [17]. Cirrhotic or non-cirrhotic, naïve, and treatment-experienced patients also showed high SVR12 rates [17].

Curry et al. conducted a multicenter, open-label trial on 268 patients randomly assigned to one of three arms: SOF/VEL + RBV once daily for 12 weeks; SOF/VEL once daily for 24 weeks; or SOF/VEL once daily for 12 weeks [15]. SVR rates were 83% (95% CI, 74-90) in patients treated with SOF/VEL for 12 weeks, 94% (95% CI, 87 to 98) in those treated with SOF/VEL plus RBV for 12 weeks, and 86% (95% CI, 77 to 92) in those treated with SOF/VEL for 24 weeks [15]. Therefore, all three treatment groups with rates of SVR significantly superior to the expected spontaneous HCV clearance rate of 1% at 12 weeks after therapy (p<0.001 for all three comparisons) reached the adjusted primary efficacy endpoint [15]. In addition, SVR rates were also successfully high in patients with chronic HCV infection and decompensated cirrhosis mainly due to decreases in bilirubin and an increase in albumin [15].

Pisaturo et al. conducted meta-analysis research in naïve individuals with chronic HCV infection and mild fibrosis [18]. They found that a SOF/VEL single-tablet regimen without RVB is very effective in chronic, non-cirrhosis HCV patients (SVR12=98%) and in HCV patients without advanced hepatic-fibrosis (SVR12=96%) [18].

All the authors mentioned above agreed that in chronic HCV patients with genotypes 1-6, including treatment-experienced and cirrhotic individuals, the SOF/VEL therapy combination was highly successful. Therefore, SOF/VEL single tablet results to be effective against all HCV genotypes with minimal risk of resistance if taken for 12 or 24 weeks.

The treatment of hepatitis C virus genotype 3 

Genotype 3 (GT3) is the second most common hepatitis C virus (HCV) genotype globally, accounting for 18% of all HCV infections in adults [19]. However, there are significant geographical differences in rates; GT3 is most common in South Asia (67%), with 54% and 79% in India and Pakistan, respectively [19]. GT3 is also common in Australia (36%) and Tropical Latin America (30%), as well as Western Europe (29%) [19]. These figures show that HCV GT3 infection affects many people and requires care since it increases the risk of hepatic steatosis, hepatic fibrosis and cirrhosis development, and hepatocellular cancer [19].

Pegylated-interferon (Peg-IFN) + ribavirin (RBV) was previously the treatment standard for HCV GT3 [20]. However, current guidelines such as those of the European Liver Studies Association (EASL) recommend either velpatasvir + sofosbuvir + ribavirin (VEL + SOF ± RBV) or daclatasvir (DCV) + SOF ± RBV as first-line therapy, dependent on the therapy experience and cirrhotic status with the RBV administration [20]. Initially, most direct-acting antivirals (DAAs) were developed using genotype 1 (GT1) replicon models. However, full-length GT1 and genotype 2 (GT2) HCV genomes have only recently been capable of replicating the whole virus life cycle in vitro [21]. Consequently, the first protease and NS5A inhibitors showed relative inadequacies in treatment results with GT3 compared to GT1 and GT2 [14]. In addition, GT3 is a genotype that is harder to cure than GT1 or GT2 [22]. Because the biology of GT3 differs from that of GT1, with faster progression, steatosis, and more significant risks of cirrhosis and primary liver cancer, the best treatments are needed [23, 24]. 

Fathi et al. focused on a subset of the data comprising existing HCV GT3 therapy [14]. They assessed the efficacy of present and future HCV treatment regimens [14]. These treatments included licensed DAAs such daclatasvir (DCV), elbasvir (EBR), grazoprevir (GZR), pegylated interferon (Peg-IFN), ledipasvir (LDV), ombitasvir (OBV), sofosbuvir (SOF) or SOF-containing, velpatasvir (VEL), as well as new, unlicensed treatments for HCV GT3, such as glecaprevir (GLE), pibrentasvir (PIB), MK-3682 (uprofosbuvir), ruzasavir (RZR) and vocilaprevir (VOX) [14]. The analysis examined combinations of these medicines with and without RBV [14]. For treating GT3 infections, the authors discovered that regimens incorporating newer DAAs (pibrentasvir + glecaprevir or grazoprevir + ruzasvir + uprofosbuvir) are more successful than those containing older DAAs [14]. In addition, the study shows that DAA regimens can treat GT3 infection instead of Peg-IFN-based treatments [14].

Ahmed et al. concluded in his meta-analysis of 1427 patients who took either SOF/VEL or SOF/VEL + RBV that the use of RBV was significantly beneficial among GT3 patients than in other genotypes [17]. It resulted in a relapse risk (RR) of 0.89, 95% confidence interval (CI) [0.80, 0.99], p=0.04, in 132 patients [17]. In patients with GT3 HCV and the role of RBV, Berden et al. studied the most effective DAA regimen among 3415 patients [25]. The highest rates of sustained virology response (SVR) were estimated among non-cirrhotic patients receiving SOF+VEL with RBV 99% (95% CI, 90%-100%) and without RBV 97% (95% CI, 95%-99%), SOF + DCV + RBV (96% (90% CI, 92%-99%) and SOF+ Peg-IFN + RBV (95%; 95% CI, 91%-98%) [25]. For cirrhotic patients, the highest levels of SVR were estimated for SOF + VEL for 24 weeks (96%; 95% CI, 92%-99%), in the group of SOF+ DCV + RBV for 24 weeks (94%; 95% CI, 87%-98%) and in patients who received SOF + VEL + RBV for 12 weeks (94%, 95% CI, 86%-98%) [25]. Ribavirin improves efficacy in both cirrhotic and noncirrhotic individuals (odds ratio, 2.6-4.5) [25].

In light of the research above, the authors have shown that the treatment of RBV with SOF-containing patterns, including newer DAA in HCV infection in GT3 patients, is more effective than previous regimens. Furthermore, when RBV adds to SOF/VEL, HCV GT3 demonstrated a greater SVR. However, its efficacy when combined with novel DAAs remains a matter of debate.

Safety of the treatment of chronic hepatitis C virus infection 

Since Peg-IFN alfa plus ribavirin (P+R) is a definitive treatment for chronic hepatitis C virus (HCV), it yields a high sustained virologic response (SVR) but with more significant side effects and poor tolerance, resulting in a suboptimal SVR rate [26]. On the other hand, sofosbuvir (SOF)-containing regimens produce better SVR rates and fewer side effects than P+R regimens, according to several randomized controlled trials (RCTs) [26].

Fan et al. analyzed eighteen randomized controlled studies (RCT) studying the safety of sofosbuvir-containing regimen versus pegylated interferon + ribavirin (P+R) on 2975 patients with varying therapy duration, regimens, therapy history, cirrhotic and non-cirrhotic [26]. The patients took a SOF-containing regimen or a P+R regimen [26]. In addition, safety-related adverse events (AE) are tested [26]. For the overall AEs, the P+R regimen was 97.1% (95% confidence interval (CI): 94.2%-98.8%), which was significantly higher than all sofosbuvir-containing regimens [26].

According to Zignego et al., 1567 patients in a series of phase III clinical trials entitled ASTRAL (ASTRAL-1, ASTRAL-2, ASTRAL-3, ASTRAL-4, and ASTRAL-5) were assessed for the safety sofosbuvir/velpatasvir (SOF/VEL) and ribavirin (RBV) [27]. Patients who received SOF/VEL + RBV had a higher rate of side effects overall and a substantially higher rate of certain events known to be associated with RBV therapy than those who received SOF/VEL alone [27]. When used with amiodarone, SOL/VEL might cause severe bradycardia [27]. Other drugs reduce SOF/VEL efficiency (antacids and proton pump inhibitors, some anticonvulsants, anti-mycobacterial, and chemotherapy) [27].

Stokes et al. conducted a systemic review and meta-analysis studying the side effects of sofosbuvir/ledipasvir/ribavirin (SOF/LDV/RBV) and sofosbuvir/ledipasvir (SOF/LDV) in 1179 patients with chronic HCV genotype 1 (GT1) [28]. Patients who got SOF/LDV/RBV experienced considerably more adverse events than those who only received SOF/LDV [28]. When comparing LDV/SOF vs. LDV/SOF/RBV, the pooled relative risk of any adverse event (AE) was 0.11 (95% CI: 0.04-0.29) [28]. There were differences in adverse effects linked with ribavirin, such as fatigue/asthenia, rash, irritability, cough/bronchitis, and anemia [28]. There were four serious adverse events linked to study therapy, all of which occurred in the SOF/LDV/RBV groups. Hemolytic anemia, nonfatal myocardial infarction, morbilliform rash, and cardiac arrest were among the complications [28].

Finally, SOF/VEL is safer with minimal side effects than with Peg-INF alpha and ribavirin. Nevertheless, caution is required to avoid drug-to-drug interactions (DDI) in the case of other concomitant treatments with SOF/VEL.

Duration of retreatment in chronic hepatitis C virus patients with relapse

Patients treated for shorter durations (four-eight weeks) with all-oral non-structural protein 5A (NS5A) direct-acting antiviral (DAA) had higher re-treatment sustained virologic response (SVR) rates than those initially treated for longer durations (10-12 weeks) [29]. The brief therapy ends in treatment failure owing to the treatment-emergent hepatitis C virus (HCV) NS5A resistance-associated variants (RAVs) [30].

Izumi et al. evaluated in an open-label study the efficacy and safety of sofosbuvir-velpatasvir (SOF/VEL) plus ribavirin (RBV) for 12 or 24 weeks [29]. The study involved 117 Japanese patients with genotype 1 hepatitis C virus (HCV) infection who are treated before with NS5A inhibitor (DAA)) or genotype 2 HCV infection with any DAA-containing regimen [29]. SVR rates were higher with 24 weeks versus 12 weeks of treatment [29]. In the 12 and 24 weeks treatment groups, 82% (47/57; 95% confidence interval (CI) 70%-91%) and 97% (58/60; 95% CI 88%-100%) of patients achieved SVR, respectively [29]. In comparison, the difference in SVR rates for the treatment groups was statistically significant (24 weeks vs. 12 weeks for all patients, p=0.023) [29]. 95% of the previously treated patients with NS5A inhibitor-based DAA for a longer duration (12-14 weeks) had NS5A or non-structural protein 5B (NS5B) resistant associated substitutions (RASs) at baseline [29]. These RASs do not have any negative effect on the treatment results [29]. Thus SOF/VEL + RBV is highly influential among this population of longer retreatment duration (24 weeks) [29]. 

Ruane et al. conducted an open-label trial to assess the efficacy of sofosbuvir/velpatasvir/vocilaprevir (SOF/VEL/VOX) for 12 weeks in chronic HCV patients who previously did not achieve SVR receiving SOF/VEL containing regimen [31]. They studied 31 cirrhotic or non-cirrhotic patients with different HCV genotypes 1-5 (GT1-5) [31]. These patients received SOF/VEL/VOX for eight weeks or SOF/VEL for 12 weeks [31]. At baseline, 32% of patients had NS5A resistance‐associated substitutions (RASs), and 26% had nonstructural protein 3 (NS3) RASs [31]. No patients had both NS5A and NS3 RASs [31]. With the re-treatment of SOF/VEL/VOX for 12 weeks, 31 of 31 patients with chronic HCV infection had an SVR rate of 100% (95% CI: 89% to 100%) in patients who had not obtained an SVR with earlier SOF and VEL exposure [31]. For NS5A inhibitor-experienced patients, a 12-week treatment of SOF/VEL/VOX is an authorized salvage treatment [31]. Few patients who failed therapy with SOF/VEL/VOX for eight weeks developed treatment-emergent resistance-associated substitutions (RASs) (one of 23 virologic failures), implying that re-treatment with the same regimen for a longer period of time may be more effective [31].

The results further support the use of SOF/VEL-containing regimen either with ribavirin (24 weeks) or VOX (12 weeks) as a salvage regimen for patients who have failed prior therapy with NS5A inhibitor‐containing regimens. With a longer re-treatment duration, HCV resistance will be diminished.

Study characteristics

Our Literature review includes patients from 11 studies. Table 1 shows the characteristics and outcomes of the included studies.

**Table 1:** Study characteristics SVR12: Sustained virologic response at 12 weeks after treatment is completed; RCT: Randomized Controlled Trial; S: Sofosbuvir; V: Velpatasvir; HCV: Hepatitis C Virus; GT: Genotype; DAAs: Direct-acting antivirals; D: Daclatasvir; VOX: Voxilaprevir; PIB: Pibrentasvir; GLE: Glecaprevir; GZR: Grazoprevir; RZR: Ruzasvir; MK-3682: Uprisofbuvir; GT3: Genotype 3; RBV: Ribavirin; P: Pegylated-interferon; S+V+(RBV): Sofosbuvir combined with velpatasvir (with or without ribavirin); S+RBV+( P): Sofosbuvir combined with ribavirin (with or without pegylated- Interferon); S+L+(RBV): Sofosbuvir combined with ledipasvir (with or without ribavirin); S+V+(RBV): Sofosbuvir combined with velpatasvir (with or without ribavirin); S+D+(RBV): Sofosbuvir combined with daclatasvir (with or with our ribavirin); AEs: Adverse events; NA: Not applicable; L: Ledipasvir; NS5A: Nonstructural protein 5A

Author	Year	Study Design	Sample Size	Treatment	Treatment Duration	SVR12 rate	Outcome
Feld et al. (1)	2015	RCT	740	S+V	12 weeks	99%	Treatment with S+V was highly effective in patients with HCV GT 1, 2, 3, 4, 5, and 6.
Fathi et al. (14)	2017	Systemic Review	2,106	Newer DAAs: S + D, S+V, S +V +VOX, PIB + GLE, GZR + RZR + MK-3682	12 weeks	>95%	Newer DAAs regimens are more effective in treating HCV GT3 infections than those containing older DAAs regimens.
Curry et al. (15)	2015	Open-label study	268	S+V+(RBV)	12 or 24 weeks	83%-94%	Treatment with S+V with or without RBV for 12 or 24 weeks resulted in a high efficacy rate in patients with HCV infection and decompensated cirrhosis.
Ahmed et al. (17)	2018	Meta-analysis	1,427	S+V+(RBV)	12 weeks	>97%	S+V regimen is highly effective in chronic HCV GT 1, 2, 4, 5, and 6 patients. Adding RBV to the S+V regimen improved the SVR12 rate of HCV GT3 patients significantly.
Mariantonietta et al. (18)	2019	Meta-analysis	6,453	S+V	12 weeks	98%	S+V regimen is suitable for all stages of liver disease.
Berden et al. (25)	2017	Systemic Review and Meta-analysis	3415	S+V+RBV S+V S+D+RBV S+P+RBV	12 weeks	95%-99%	The analysis indicated that RBV significantly increases the SVR rate in HCV GT3 patients.
Fan et al. (26)	2018	RCT	2,975	S+V+(RBV), S+RBV+( P), S+L+(RBV), S+V+(RBV,) S+D+(RBV), P+RBV	12 or 24 weeks	NA	The side effects of P+RBV were 97.1%, which was significantly higher than all sofosbuvir-containing regimens.
Zignego et al. (27)	2018	Clinical Trials	1,567	S+V+RBV	12 weeks	NA	S+V+RBV regimen has a higher rate of AEs than S+V alone, which is known to be associated with RBV therapy.
Stokes et al. (28)	2017	Systemic Review and Meta-analysis	1,179	S+L+(RBV)	12 weeks	NA	In comparison to RBV, the S+V regimen is safer with fewer adverse effects.
Izumi et al. (29)	2018	Open-label study	117	S+V+RBV	12 or 24 weeks	82%-97%	S+V+RBV for 24 weeks was more effective than 12 weeks in Japanese patients with chronic HCV infection who previously failed treatment with a DAA.
Ruane et al. (31)	2019	Open-label study	31	S+V+VOX	12 weeks	100%	The results support the use of S+V+VOX for 12 weeks as a salvage regimen for patients who have failed prior therapy with NS5A inhibitor-containing regimens.

Limitations

This literature review includes many randomized control trials and meta-analyses. It has few limitations, comprising articles published in the English language only within the last five years. The findings are in part based on the results of observational studies in a small sample sizes.

## Conclusions

We studied the efficacy of sofosbuvir/velpatasvir (SOF/VEL) in the treatment of patients suffering from chronic hepatitis C virus (HCV) infection. Our literature review, which is consistent with the American Association for the Study of Liver Diseases (AASLD) guidelines, shows that 12 weeks of SOF/VEL regimen effectively treats chronic HCV-infected individuals with compensated cirrhosis and without cirrhosis for any genotype. Furthermore, SOF/VEL is also successful in HCV-infected patients with decompensated cirrhosis and ribavirin ineligible if treated for 24 weeks. To establish the usage of SOF/VEL globally, future well-designed clinical studies with a large sample size will be necessary. Researchers should review the cost-effectiveness of these medications because they are considered high-priced.
